# HLA antibody testing: a tool to facilitate not to prevent organ transplantation

**DOI:** 10.1111/j.1744-313X.2008.00800.x

**Published:** 2008-08

**Authors:** Frans H J Claas

## Abstract

The introduction of very sensitive HLA antibody screening assays has destroyed the old dogma that pre-existence of donor specific HLA antibodies in the patient is a contra-indication for transplantation. The challenge is now to reach consensus on the parameters which predict the clinical relevance of donor specific HLA antibodies. Antibody screening assays should not only be used to prevent transplantation of patients with donor specific antibodies but also to facilitate transplantation of highly sensitized patients, both by defining acceptable HLA mismatches and non-detrimental donor specific HLA antibodies.

In this issue, David Eckels discusses the clinical implications of solid-phase human leucocyte antigen (HLA) antibody testing for organ transplantation (Eckels, 2008).

Although the manuscript was considered too controversial according to American standards, it is an excellent basis for discussions on this important topic and, hopefully, will stimulate international collaboration aiming at solving the many issues, which are not supported by solid scientific evidence yet. Actually, many of the points raised in this discussion paper are not controversial at all and some of them even common use in many transplantation programs in Europe and particularly within Eurotransplant.

Since the introduction of very sensitive luminex-based assays by commercial companies, both HLA laboratories and clinicians are confused. They were all educated by the old dogma that donor-specific antibodies are always a contraindication for transplantation (Patel & Terasaki, 1969) and many of them immediately gave a similar value to antibodies detected by these newly developed assays. However, it is clear that this dogma is not valid anymore, and the pretransplant assessment of donor-reactive HLA-specific antibodies should rather be considered as a parameter for the risk of adverse complications after transplantation (Gebel *et al*., 2003). Within Eurotransplant the standard cross-match is still the original complement-dependent cytotoxicity (CDC) assay, known to be clinically relevant. Many American colleagues consider this approach as not ethical (even reviewers of our papers make such remarks) but transplant results in (highly) sensitized patients are at least as good in Europe compared to the USA (Doxiadis *et al*., 2005). On the other hand, complicated and very expensive desensitization programs are applied by different centres in the USA in order to transplant ‘sensitized’ patients after removal of antibodies, which are not detectable in CDC and, until recently, not even monitored in Europe. Desensitization is probably useful for a proportion but certainly not for all patients included in these programs.

A recent retrospective study in highly sensitized patients, transplanted on the basis of a negative CDC cross-match, showed that the presence of non-complement fixing donor-specific antibodies detected by luminex is associated with a (treatable) rejection in only a subpopulation of patients while many patients with donor-specific antibodies only detectable in luminex have an excellent long-term kidney graft survival (van den Berg-Loonen *et al*., 2008).

The challenge is, as stated by David Eckels, to discriminate clinically relevant from non-clinically relevant antibodies. In order to do so, it is essential to define the actual specificities of the antibodies present in the serum of a patient. Preferentially, one should be able to explain these specificities by a previous sensitizing event in the history of the patient. In the current era with our increased knowledge of the antibody epitopes present on the HLA antigens (Duquesnoy, 2006; El-Awar *et al*., 2007), this is feasible. However, the problem is that many centres do accept all specificities generated by the computer programs linked to the commercial screening assays without evaluating whether these antibody specificities make any sense, immunologically speaking.

As already stated by David Eckels, luminex-based assays from different companies may generate different antibody specificities, which is clearly an argument to look critically at the results. One of the reasons why not all antibody specificities are relevant is the fact that these assays are based on antibody binding to isolated HLA molecules, which may have a different conformation than the HLA molecules naturally expressed on the cell membrane of the donor organ.

But even the presence of well-defined donor-specific HLA antibodies is certainly not always a contraindication for transplantation. On one hand, these antibodies may lead to hyperacute or early acute humoral rejection but, on the other hand, they may be associated with no negative clinical effect (van den Berg-Loonen *et al*., 2008) or even an enhanced graft survival (Koka *et al*., 1993).

The challenge is to preassess the risk associated with the presence of donor-specific antibodies and to use this knowledge for donor selection and/or the immunosuppressive policy around or after transplantation.

I do not agree with the statement of David Eckels that we should drop the CDC test as, in contrast to antibodies detected by the other very sensitive assays, a positive CDC cross-match due to donor HLA-specific IgG antibodies is known to be clinically relevant in the majority of the cases. As long as we do not agree on the relevance of all the other assays, a standard CDC cross-match is helpful to prevent transplantation of patients with detrimental donor-specific HLA antibodies. The next necessary step is to start international collaboration to determine the clinical relevance of all the different types of antibodies.

Although several reports show the presence of HLA-C, -DQA, -DQB, -DPA and -DPB antibodies in potential transplant recipients (i.e. Duquesnoy *et al*., 2008), no systematic analysis of their clinical relevance has been performed and as a consequence many completely different local policies are used in patients with these types of antibodies.

Both the expression of the target molecules in the transplanted organ and the titre and immunoglobulin class (or subclass) may play a determinative role in this aspect.

The basis of our decision-making should be antibody specificity and not percentage panel reactive antibodies (%PRA) as it has been for years. The old definition of percentage PRA (just based on antibody reactivity against a panel) must disappear and percentage PRA should be based on antibody specificities in combination with the frequency of the target antigens in the donor population. Actually, such an algorithm has been introduced in Eurotransplant.

In the future, one should be able to calculate two types of PRA: first, percentage PRA on the basis of antibodies, which are a contraindication for transplantation, and second, percentage PRA on the basis of antibodies, which should be taken into consideration with respect to the choice of immunosuppressive treatment if the patient underwent transplantation despite the presence of these antibodies.

Although knowledge of antibody specificities is important, even more important is a reliable definition of acceptable mismatches, those HLA antigens towards the patient never made a (potentially detrimental) antibody response. Donor selection based on acceptable mismatches has shown to be a very efficient tool to enhance transplantation of highly sensitized patients within Eurotransplant (Claas *et al*., 2004). If the future definition of acceptable mismatches is based on very sensitive luminex assays, one can even consider transplanting highly sensitized patients without performing a pretransplant cross-match provided that donor and recipient are typed at a high resolution level, thereby excluding a possible effect of allele-specific HLA antibodies.

It is clear that the introduction of solid-phase HLA antibody tests has had a tremendous impact on the field of histocompatibility testing. What is currently lacking is consensus on the relevance of the different types of donor-specific antibodies for donor selection (which antibodies are a contraindication for transplantation) and for immunosuppressive treatment (which antibodies are associated with treatable early acute humoral rejection).

It is essential to use the results of antibody screening assays not only to prevent transplantation of patients but to facilitate transplantation of (highly) sensitized patients on basis of acceptable mismatches or in the presence of clinically non-detrimental donor-specific HLA antibodies.

Routinely monitoring the appearance of donor-specific antibodies after transplantation may be an additional tool to prevent loss of transplanted organs provided that adequate treatment is available (Mao *et al*., 2007).

However, we should not forget that the best policy is prevention of antibody formation. HLA matching, and especially matching of donor and recipient for the antibody epitopes present on the HLA molecules (Dankers *et al*., 2004), will prevent that many retransplant candidates will finally end up as highly sensitized patients with very extensive antibody profiles in the current solid-phase assays.
